# Gradient‐Integrated High‐Strength and High‐Toughness Cellulose Triboelectric Materials for Enhanced Charge Storage and Transport

**DOI:** 10.1002/advs.78778

**Published:** 2026-11-10

**Authors:** Pinle Zhang, Yanhao Xu, Huancheng Huang, Neng Xiong, Xinke Yu, Xinyue Nong, Yingping He, Ruizhi Li, Shuangfei Wang, Xinliang Liu

**Affiliations:** ^1^ Guangxi Key Laboratory of Clean Pulp & Papermaking and Pollution Control School of Light Industry and Food Engineering Guangxi University Nanning China

**Keywords:** cellulose, integrated triboelectric materials, interface engineering, self‐powered sensor, triboelectric nanogenerator

## Abstract

The rapid advancement of wearable electronics places ever‐increasing demands on the electrical and mechanical properties of materials. Cellulose offers renewability, low cost, and high durability for green wearables, but its weak electron‐donating ability and poor charge‐transport efficiency limit triboelectric performance. Interfacial mismatch between the electrode and triboelectric layers also induces charge dissipation, further aggravating this problem. Inspired by the Janus structure found in sea urchin spines, a gradient‐integrated triboelectric material (MWCNT/T‐CNFs) was fabricated via vacuum‐assisted layer‐by‐layer filtration. The resulting gradient electric field forms a highly compatible interface between the triboelectric layer and the electrode layer, providing a low‐impedance path that effectively suppresses charge dissipation. Consequently, the material achieves a surface charge density of 20.5 nC cm^−2^ (931.8% that of pure T‐CNFs) and excellent cycling stability (8044 cycles). The integrated interface offers strong adhesion, far outperforming conventional electrode tapes, while the gradient design alleviates stress concentration, yielding a tensile strength of 126.33 MPa and toughness of 9.11 MJ m^−3^(535.9% that of pure T‐CNFs). The material also exhibits good water stability and high‐temperature resistance. This study addresses the key limitations of low surface charge density and interfacial mismatch in cellulose materials, providing a solid foundation for interface design in wearable electronics.

## Graphical abstract

Inspired by the gradient structure of sea urchin spines, a gradient‐integrated cellulose/multi‐walled carbon nanotube (MWCNT) triboelectric material is constructed. A compatible interface and built‐in electric field synergistically suppress charge dissipation, achieving 20.5 nC cm^−2^ surface charge density, 8044‐cycle stability, 126.33 MPa tensile strength, and 9.11 MJ m^−3^ toughness, providing a foundation for interfacial design in green self‐powered wearable electronics.

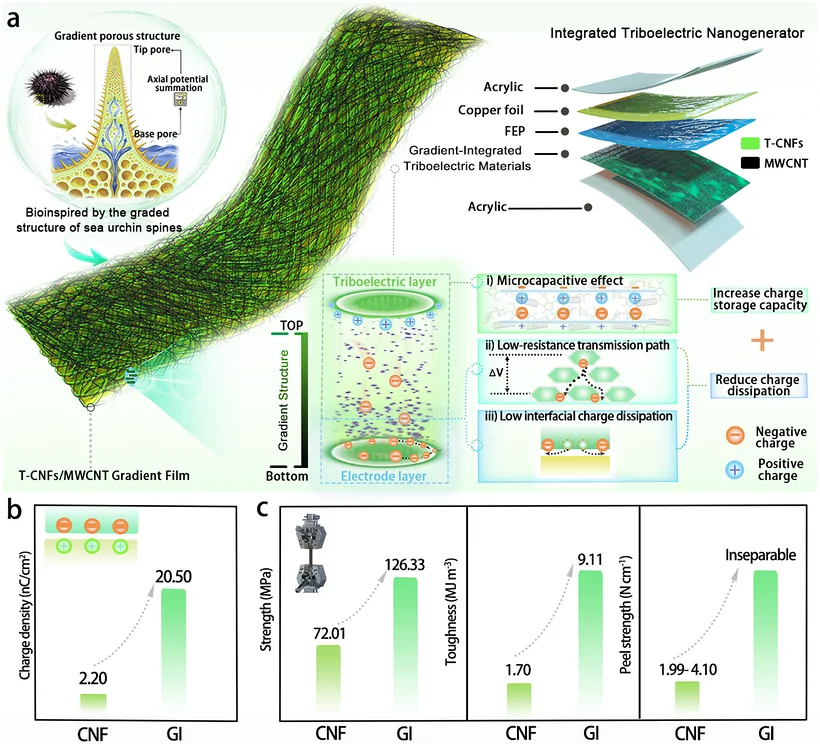

## 1 Introduction

Rapid advancements in wearable electronic devices impose increasingly stringent demands on flexibility, lightweight construction, and mechanical durability. In particular, stable operation under repeated bending and twisting has become a critical criterion for practical application [1, 2]. Triboelectric nanogenerators (TENGs), which convert ambient mechanical energy into electricity, offer a promising route to flexible, self‐powered wearable electronics, owing to their diverse material availability, adaptable structural designs, and dual functionality in energy harvesting and self‐powered sensing [3, 4]. The performance of TENGs is largely determined by the selection of triboelectric materials, among other factors. However, conventional TENGs typically employ triboelectric layers composed of non‐biodegradable or expensive materials, such as polyvinylidene fluoride (PVDF), polytetrafluoroethylene (PTFE), polydimethylsiloxane (PDMS), polyamide (PA), or copper [5, 6, 7]. Although TENGs based on conventional materials exhibit satisfactory output performance, they suffer from significant drawbacks in terms of environmental friendliness, biosafety, and sustainability. Cellulose, the most abundant natural biopolymer, has emerged as a promising alternative to traditional triboelectric layer materials due to its renewability, non‐toxicity, excellent mechanical properties, and electron‐donating ability conferred by abundant surface hydroxyl groups [8, 9]. However, the intrinsically limited polarization capability of native cellulose restricts its charge capture capacity, leading to suboptimal output performance [10, 11, 12]. Therefore, increasing the surface charge density of cellulose‐based TENGs has become a critical challenge in this field.

To address these performance bottlenecks, various enhancement strategies have been proposed, including surface modification (e.g., physical doping, chemical modification), structural optimization, and charge injection [13, 14]. Among these, doping with inorganic fillers is widely adopted due to its simplicity and effectiveness. The underlying mechanism is to increase the dielectric constant of the triboelectric material, thereby enhancing surface charge density. However, current doping strategies face a critical trade‐off: excessive conductive fillers tend to agglomerate and form conductive pathways, exacerbating triboelectric charge dissipation; conversely, insufficient doping fails to significantly improve charge transfer efficiency. Moreover, filler content substantially affects mechanical properties. Using carbon nanotubes as an example, low filler loading yields limited mechanical reinforcement, while excessive loading induces agglomeration and stress concentration, reducing mechanical strength and toughness. This compromises the flexural durability required for wearable devices under repeated bending and twisting. An effective resolution to this trade‐off remains elusive.

Beyond the intrinsic limitations of material modification, cellulose‐fiber‐based triboelectric layers face another major constraint: interfacial charge dissipation arising from the mechanical mismatch between the fiber network and the electrode layer [15, 16]. Cellulose nanofibers (CNFs) possess a high aspect ratio and good flexibility, enabling the formation of three‐dimensional porous networks that provide excellent mechanical compliance. However, this fiber network exhibits low surface energy on a macroscopic scale and limited interfacial contact with rigid electrodes (e.g., metal films), resulting in weak bonding. Typically, electrodes are made of high‐modulus or low‐strength rigid materials, whereas the fiber‐based triboelectric layer is flexible and porous. This mechanical disparity not only impedes charge transfer from the triboelectric layer to the electrode, causing dissipation due to interfacial barriers, but also promotes interfacial separation or localized delamination under repeated bending and stretching. Such instability severely compromises the electrical output stability and service life of the TENG [17]. Therefore, enhancing the interfacial bonding strength between the fiber network and the conductive layer, while preserving the network's inherent flexibility, has become a key challenge for improving the long‐term stability of cellulose‐based TENGs.

To address two major challenges—the trade‐off between charge capture and dissipation, and mechanical incompatibility at the fiber‐electrode interface—this study introduces a gradient integration (GI) design strategy inspired by the gradient structure and function of sea urchin spines. The GI triboelectric material is fabricated using a simple hierarchical filtration method, employing TEMPO‐oxidized cellulose nanofibers (T‐CNFs) as the substrate and multi‐walled carbon nanotubes (MWCNTs) as the conductive filler. The concentration gradient generated by MWCNTs forms ‘micro‐capacitor’ units from top to bottom, enhancing charge capture efficiency. Concurrently, the gradient electric field mitigates localized electric field concentrations and suppresses charge dissipation through tunnelling and leakage. The dense conductive network at the bottom provides a low‐resistance pathway for induced charges, thereby reducing transmission losses. The integration of the triboelectric layer with the conductive electrode layer fundamentally eliminates interfacial charge dissipation and delamination caused by weak bonding and mechanical mismatch in conventional TENGs, ensuring stable output under complex mechanical deformations. This work successfully resolves the inherent conflict between charge capture and dissipation in conventional doping strategies, as well as the degradation of electrical and mechanical properties resulting from interfacial mechanical mismatch, providing a new theoretical and technical foundation for high‐performance, green, and sustainable triboelectric materials.

## 2 Results and Discussion

### 2.1 Design of Triboelectric Materials

The gradient‐pore structure, modeled on sea urchin spines, offers two synergistic benefits. Mechanically, it achieves an optimal balance between strength and toughness. Electrically, the pore‐size gradient generates a spatial electric potential field that facilitates charge separation and enhances output, thereby raising the overall mechanical‐to‐electrical energy conversion efficiency. Inspired by this natural model, a gradient‐integrated triboelectric material is constructed via a simple layered filtration method. The concentration gradient of the conductive filler not only generates a microcapacitive effect to enhance charge trapping, but also creates a gradient electric field that counteracts local field concentration, thus curtailing charge dissipation from interfacial tunnelling and leakage. In parallel, the compact conductive network in the bottom layer establishes a low‐loss channel for charge transport to the electrode, effectively reducing transmission losses. As a result, the holistic architecture circumvents the charge leakage/dissipation challenges typically associated with mismatched triboelectric/electrode interfaces (Figure 1a).

**FIGURE 1:**
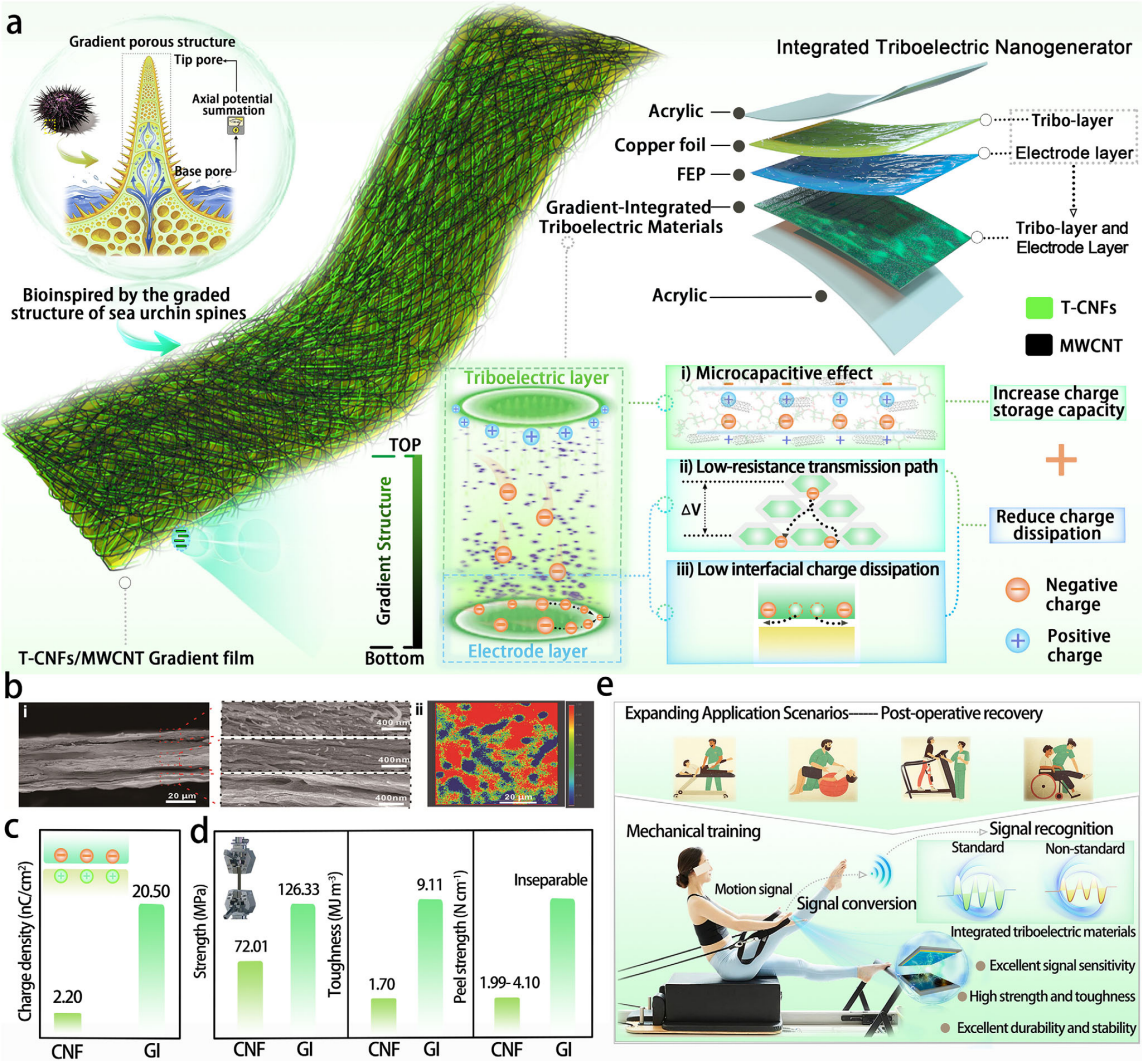
Design of the GI triboelectric material. (a) Schematic illustration of the structural principles of gradient‐integrated triboelectric materials, inspired by the architecture and function of sea urchin spines. (b) Cross‐sectional structural features: (i) SEM image. (ii) 2D Raman spectrum. (c) High surface charge density. (d) Superior mechanical properties. (e) Application in self‐powered wearable rehabilitation training for motion sensing.

The gradient structure is evident in both the cross‐sectional SEM image and the 2D Raman spectrum (Figure 1b). This structure simultaneously enhances charge storage capacity and enables low‐dissipation integrated charge storage and transport, resulting in a high surface charge density (Figure 1c). Additionally, it effectively mitigates stress concentration, significantly enhancing the mechanical strength and toughness of cellulose‐based triboelectric materials, whilst its integrated structural design confers resistance to impact‐induced delamination (Figure 1d). The combination of high mechanical strength, toughness, impact resistance, and high surface charge density endows this material with excellent responsiveness and stable signal output for the detection of typical movements during stretching and rehabilitation exercises (Figure 1e).

### 2.2 Structural Analysis of GI Triboelectric Materials

In the GI triboelectric material design, the high electrostatic affinity of T‐CNFs ensures uniform MWCNT dispersion [18]. Through π‐π stacking between T‐CNFs' carboxyl groups and the MWCNT carbon framework, along with hydrogen bonding, MWCNTs wrap around T‐CNFs to form a dense, network‐like gradient structure (Figure 2a). The GI film retains excellent mechanical toughness due to electrostatic forces and π‐π interactions (Figure 2b).

**FIGURE 2:**
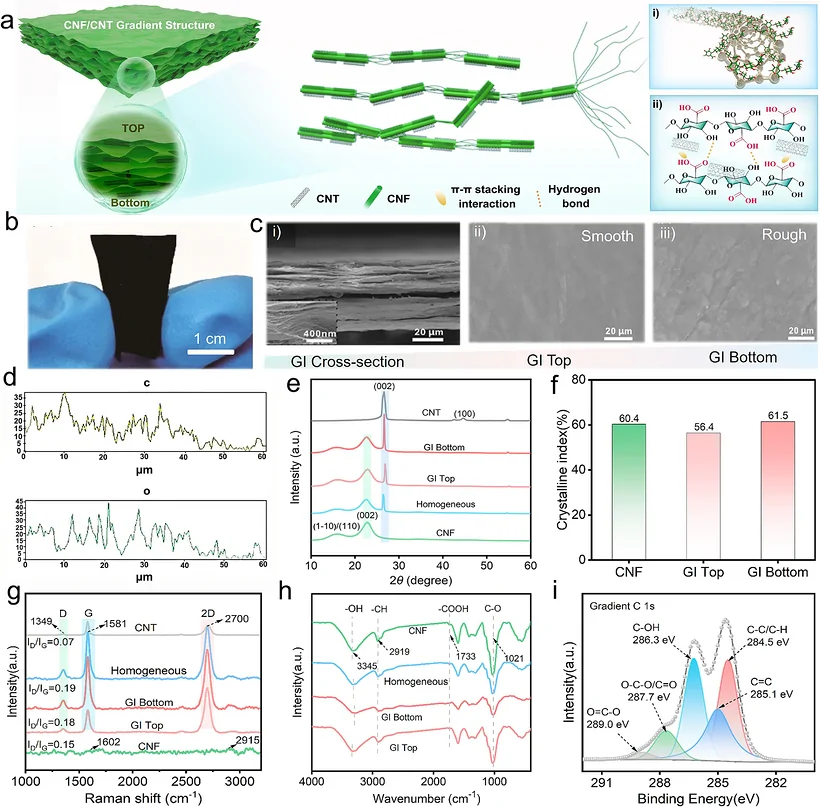
Structural analysis of GI triboelectric materials. (a) Schematic of the GI structure. (i) Schematic diagram illustrating the behavior of T‐CNFs and MWCNTs. (ii) Schematic diagram illustrating the binding mechanism of T‐CNFs and MWCNTs. (b) Photograph of the GI film. (c) Scanning electron microscope images of the GI film. (i) SEM cross‐sectional image; (ii) SEM top view; (iii) SEM bottom view. (d) SEM cross‐sectional line scan. (e) XRD patterns, (f) crystallinity, (g) Raman spectra, and (h) IR spectra of the three materials. (i) C 1s XPS spectra of the GI material.

The cross‐sectional morphology of the gradient T‐CNFs/MWCNT composite film reveals that the CNFs act as a scaffold, with MWCNTs wound around the T‐CNFs to form a highly dense network structure (Figure 2c i). The top layer of GI retains a high degree of smoothness, with the CNTs completely encapsulated by the CNF network; the bottom layer exhibits nanoscale protrusions due to CNT enrichment, but shows no obvious surface damage or voids (Figure 2c ii, iii and Figure S1). Linear mapping scans confirm a continuous increase in MWCNT concentration from top to bottom (Figure 2d). The observation confirms the formation of the gradient structure design.

XRD and Raman spectroscopy reveal differences in molecular vibrations and chemical states between the two surfaces. Characteristic peaks at 15.5° and 22.8° correspond to the (110) and (200) planes of type I cellulose (Figure 2e) [19, 20]. The crystallinity index of pristine T‐CNFs is 60.4%. Minor MWCNT doping disrupts some hydrogen bonds between cellulose chains, loosening the ordered crystalline regions and temporarily reducing crystallinity to 56.4%. With higher doping, heterogeneous nucleation on MWCNT surfaces promotes adsorption and ordered arrangement of cellulose molecules, forming a highly crystalline interfacial phase, thus increasing bottom crystallinity to 61.50% (Figure 2f). This crystallinity gradient helps the material accommodate mechanical deformation and resist stress‐concentration‐induced tearing [21]. The composite film displays characteristic Raman peaks at 1349, 1581, and 2700 cm^−1^, consistent with reported T‐CNFs/MWCNT interactions [22]. The D‐band reflects lattice defects; the ID/IG ratio indicates defect density, which is positively correlated with electron conduction efficiency. The bottom ID/IG ratio is 0.171(Figure 2g). The high MWCNT content leads to increased cellulose encapsulation and a significantly higher density of surface defects on MWCNTs, facilitating charge transport [23].

The FT‐IR spectra (Figure 2h) show that pure T‐CNFs exhibit characteristic peaks at 3345 cm^−1^ (O─H stretching), 2919 cm^−1^ (C─H symmetric stretching), 1733 cm^−1^ (C═O stretching), and 1021 cm^−1^ (C─O stretching) [24]. In the composite film, the O─H peak shifts to 3343 cm^−1^ and the C═O peak weakens, indicating hydrogen bonding between the carboxyl groups of T‐CNFs and oxygen‐containing groups or defect sites on MWCNTs [25]. Moreover, the O─H (3370 cm^−1^) and C═O (1725 cm^−1^) peak intensities are significantly reduced at the bottom of the gradient film due to high‐density MWCNT doping and cellulose encapsulation. In contrast, the top surface with low doping shows peak intensities close to those of pure T‐CNFs, confirming the successful formation of the gradient structure. XPS analysis (Figure 2i and Figure S2) reveals enhanced C─OH, C─O─C, and O─C═O peaks in the GI material compared to pure T‐CNFs, attributed to hydrogen bonding. An absorption peak at 285.1 eV corresponds to the C═C conjugated double bonds in MWCNT aromatic rings [26], confirming noncovalent π‐π stacking and hydrogen bonding between MWCNTs and T‐CNFs.

### 2.3 Mechanical Properties of GI Triboelectric Materials

The mechanical properties of triboelectric materials critically determine their application scope. For wearable tactile sensors, high sensitivity, flexibility, and stability are essential. Conventional TENGs use copper or aluminum tape bonded to the triboelectric layer as the electrode; although simple to fabricate, this approach lacks flexibility and comfort, rendering it unsuitable for wearable sensing. In contrast, GI triboelectric materials exhibit a dense, non‐peelable structure due to strong hydrogen bonding between fibers under vacuum pressure (Figure 3a,b), outperforming common conductive tapes such as those made of copper foil or copper‐nickel fibers. At 10 wt.% MWCNT content, the tensile stress and strain show the most significant increase (Figure S3). Specifically, tensile strain rises from 4.17% (T‐CNFs) to 6.61%, tensile stress from 72.01 to 91.01 MPa, and toughness from 1.70 to 3.64 MJ m^−3^. This indicates that an appropriate amount of carbon nanotubes enhances interactions within the cellulose material. The gradient‐doped film exhibits markedly improved mechanical properties, with a tensile stress increase of 126.33 MPa and a tensile strain increase of 14%, 1.75 times that of the pure T‐CNFs film (Figure 3c). The modulus decreases from 1.38 to 0.92 GPa, attributed to intermolecular hydrogen bonding and π‐ π stacking that form a more compact film [27], endowing the gradient composite with tunable strength and good flexibility. The optimal aspect ratio of the two components further promotes their intertwining, enhancing tensile properties. The GI design enables progressive stress transfer via modulus differences between layers of varying density—a highly flexible surface combined with a rigid base. This structural design creates an interfacial stress gradient between the upper and lower T‐CNFs layers, releases residual stresses within each layer, and concentrates load at higher‐stress interfaces, thereby significantly improving mechanical properties (Figure 3d–f) [28]. The resulting tensile strength outperforms existing T‐CNFs/MWCNT systems (Figure 3g) [25, 29, 30, 31, 32, 33].

**FIGURE 3:**
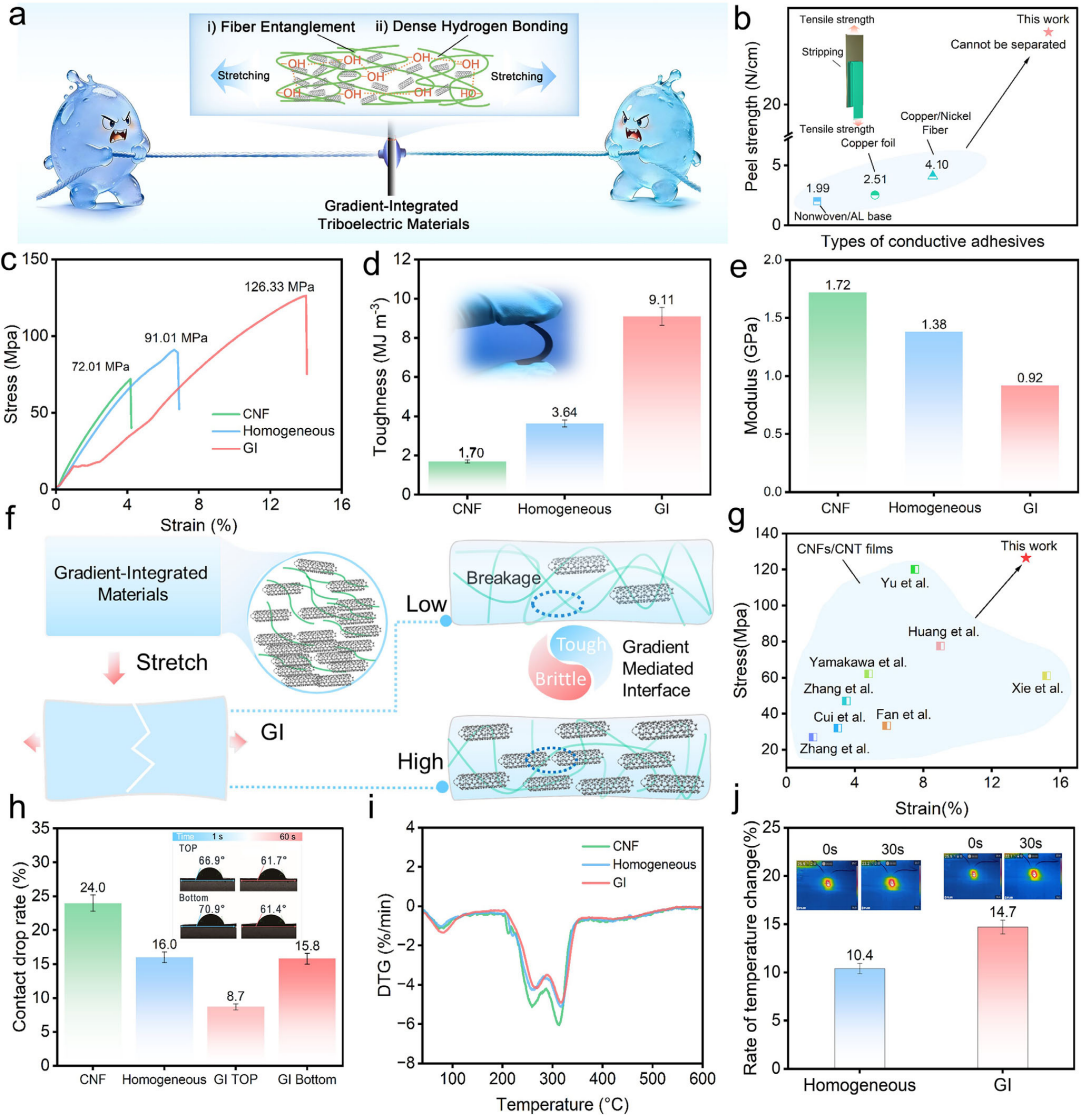
Mechanical properties of GI triboelectric materials. (a) Anti‐delamination mechanism between the triboelectric and electrode layer. (b) Comparison of anti‐delamination performance. (c) Stress–strain curves of the three materials. (d) Toughness, (e) Modulus, and (f) Schematic of the strength‐ toughness balance mechanism. (g) Mechanical properties comparison with other cellulose/carbon nanotube materials [25, 29, 30, 31, 32, 33]. (h) Water contact angle stability. (i) Second derivative of thermogravimetric curves. (j) Thermal conductivity comparison.

The wettability of triboelectric materials is critical for applications in complex environments. TEMPO‐oxidized cellulose, rich in hydrophilic groups [34], exhibits a dense, smooth surface with high barrier properties, yielding a contact angle of 56.6°. MWCNTs are superhydrophobic; an appropriate loading enhances the hydrophobicity of the composite film, whereas excessive doping creates a porous surface and reduces hydrophobicity. The gradient structure shows a top contact angle of 66.9° and a bottom angle of 70.9° (Figure S4 and Figure 3h), reflecting the hydrophilicity/hydrophobicity difference induced by the gradient MWCNT content. After 1 min of standing, the contact angles of the T‐CNFs film, uniformly doped film, and GI composite film (top and bottom) decrease to 42.1°, 61.8°, 61.7°, and 61.4°, respectively—reductions of 24.0%, 16.0%, 8.7%, and 15.8% from their initial values. The initial smooth cellulose surface absorbs water via hydrophilic groups, leading to swelling and rapid water penetration. The introduction of MWCNTs promotes cellulose adsorption onto the MWCNT surface, forming a denser surface structure. The hydrophobic nature of MWCNTs reduces the water absorption rate. Moreover, the gradient structure exhibits a lower water absorption rate than the homogeneous structure, indicating a more compact internal architecture.

The thermal stability of the materials was evaluated by TGA. The residual mass at 600°C increased from 20% for the cellulose film to over 50% for all T‐CNFs/MWCNT composite films (Figure S5), which is mainly attributed to the intrinsic non‐decomposability of MWCNTs. The DTG peak temperature increased from 310°C for pure T‐CNFs to 317°C for the homogeneous film and 319°C for the GI film (Figure 3i). The GI film exhibits the highest DTG peak temperature, indicating improved thermal stability. This improvement is attributed to the gradient MWCNT distribution, which promotes heat diffusion and delays localized heat accumulation within the cellulose matrix. The gradient structure, with its high MWCNT concentration at the bottom, promotes gradient heat diffusion, delays localized heat accumulation, and thus enhances thermal stability. Infrared thermal imaging showed that the temperature reduction rate of the gradient structure within 30 s was 14.7%, higher than the 10.4% of the homogeneous structure, confirming its superior heat diffusion capability (Figure 3j and Video S1). Overall, the GI design lays a foundation for applications in high‐temperature environments.

### 2.4 The Electrical Properties of GI Triboelectric Materials

Gradient doping creates a dielectric constant gradient. According to the series capacitor principle, the high‐ε layer experiences a low electric field, whereas the low‐ε layer experiences a high electric field, generating a non‐uniform electric field within the material. The contact potential difference (CPD) was analyzed using Kelvin probe force microscopy (KPFM). The top layer, with low carbon nanotube concentration, forms microscopic capacitors [35]; the dispersed nanotubes do not form a continuous conductive network but effectively capture triboelectric charges, resulting in a high surface potential (510 mV). Conversely, the bottom layer, with a dense conductive network from high‐concentration nanotubes, accelerates directed charge transport to the electrode and reduces local accumulation, yielding a lower surface potential (432 mV) (Figure 4a,b). These results confirm the presence of an inhomogeneous electric field between the GI film layers.

**FIGURE 4:**
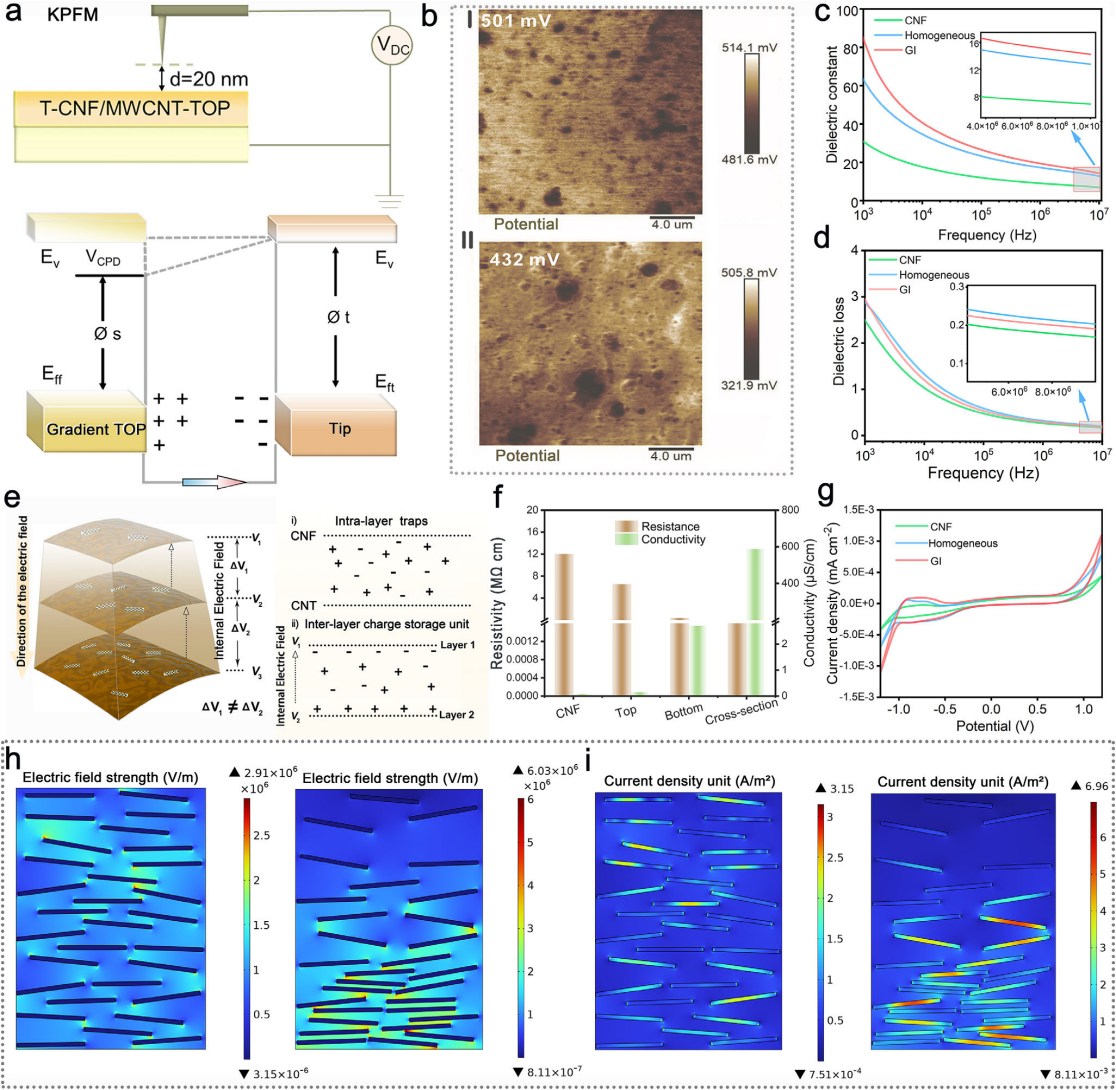
Electrical properties of GI triboelectric materials. (a) Schematic of the KPFM principle. (b) Top and bottom surface potentials measured by KPFM. (c) Dielectric constants. (d) Dielectric losses. (e) Charge storage mechanisms of the three materials. (f) Surface and bulk conductivities. (g) Cyclic C‐V curves. (h) Electric field modes and (i) current modes from COMSOL simulations (ii).

Inorganic nanofillers with high conductivity, when combined with insulating cellulose, enhance the dielectric constant through microcapacitance and interfacial polarization effects [12]. Broadband dielectric measurements (Figure 4c,d) reveal that, at a frequency of 10^5^ Hz, the dielectric constant of the uniformly doped composite film increases from 12 for pure T‐CNFs to 22, demonstrating that uniform dispersion of carbon nanotubes effectively enhances the dielectric constant. However, the resulting conductive pathways and tunneling effects induce leakage current, thereby increasing the dielectric loss from 0.48 to 0.59 [36]. By contrast, the GI film exhibits a higher dielectric constant of 26 (2.17 times that of T‐CNFs) and a dielectric loss of 0.53, which is lower than that of the uniformly doped film but slightly higher than that of pure T‐CNFs. These results indicate that the gradient architecture suppresses excessive dissipation caused by the conductive MWCNT network, rather than eliminating the intrinsic interfacial polarization loss introduced by MWCNTs. This enhancement is attributed to the formation of numerous microcapacitive units between MWCNTs and T‐CNFs, where interfacial charge accumulation generates a large number of small dipoles under an external electric field, thereby enhancing the charge storage capacity. In addition, the gradient distribution prevents the formation of a continuous conductive network across the film, while the T‐CNF‐rich layers act as insulating and charge‐blocking layers that reduce leakage current and long‐range charge migration. Consequently, the gradient architecture achieves a favorable trade‐off between enhanced polarization and suppressed dissipation, leading to improved effective charge storage [37].

Additionally, the conductivity difference induces strong Maxwell‐ Wagner interfacial polarization, causing free charges to aggregate and relax at the interfaces for efficient energy storage. Furthermore, the gradient electric field between layers alleviates local field concentration and suppresses charge dissipation caused by interface charge penetration and leakage, leading to higher charge storage efficiency (Figure 4e). The conductive graphitized MWCNTs encapsulate the insulating T‐CNFs scaffold. The resulting gradient structure exhibits high electrical conductivity (surface: 2.71 µS cm^−1^; bulk: 588.23 µS cm^−1^), enabling rapid directed charge transfer and increasing the transient current in the external circuit [38] (Figure 4f). Cyclic voltammetry (C‐V) shows that the GI film achieves the highest current density at high potentials, confirming its superior charge transport and storage capabilities (Figure 4g and Figure S6). COMSOL simulations of the internal electric field under an external field reveal that uniformly doped MWCNTs produce a relatively uniform field with a low overall strength of 4.50 × 10^6^  V m^−1^. In contrast, the GI film exhibits significant variations in the electric field modulus as the gradient changes, reaching a maximum of 6.96 × 10^6^ V m^−1^, indicating a significantly enhanced internal electric field gradient (Figure 4h). In the current mode, uniformly doped films display a uniform current distribution; the GI film's gradient electric field provides spatial sites for charge storage at the top, while the strong field at the bottom creates a low‐loss pathway for charge transport (Figure 4i).

### 2.5 Electrical Output Characteristics and Underlying Mechanisms of GI Triboelectric Materials

The positive electrode is the GI triboelectric material, and the negative electrode is an FEP film adhered to an acrylic plate with conductive adhesive; copper wires connect to the test equipment (Figure 5a). COMSOL simulations of the contact‐separation process show that a potential difference develops between the two triboelectric layers upon separation, driving electron flow through the external circuit (Figure 5b). The operating principle of GI‐TENG is illustrated in Figure 5c and Figure S7). Initially, no charge is present. Upon contact, the GI material becomes positively charged, and the FEP film becomes negatively charged. When the force is removed, to maintain the potential difference, opposite charges are induced at the electrodes, and electrons flow from the bottom to the top. Upon reapplication of force, the contact induces additional charge, and electrons flow from the top to the bottom, completing one working cycle [39]. In this working mechanism, storage and transport are governed by two distinct charge types: triboelectric charges physically trapped on the surface and within the bulk of the dielectric layers serve as the electrostatic source, while free electrons (induced charges) flowing through the external circuit generate the output current. Accordingly, the incorporation of MWCNTs forms micro‐capacitors with the cellulose nanofibers through interfacial polarization effects. At the high‐resistance end, the gradient distribution provides more micro‐capacitor units for charges, enhancing the charge capture capability. Meanwhile, during contact‐separation, owing to the integrated interfacial structure design, the triboelectric charges are driven longitudinally with low hindrance toward the low‐resistance region under the internal gradient electric field [38], thereby effectively suppressing bulk charge leakage and achieving efficient capture and low‐loss transport of triboelectric charges. Under electrostatic induction, the internal electric field gradient is enhanced, driving more induced charges to migrate toward the electrode‐contacting surface, ultimately leading to a significant enhancement in electrical output (Figure 5d).

**FIGURE 5:**
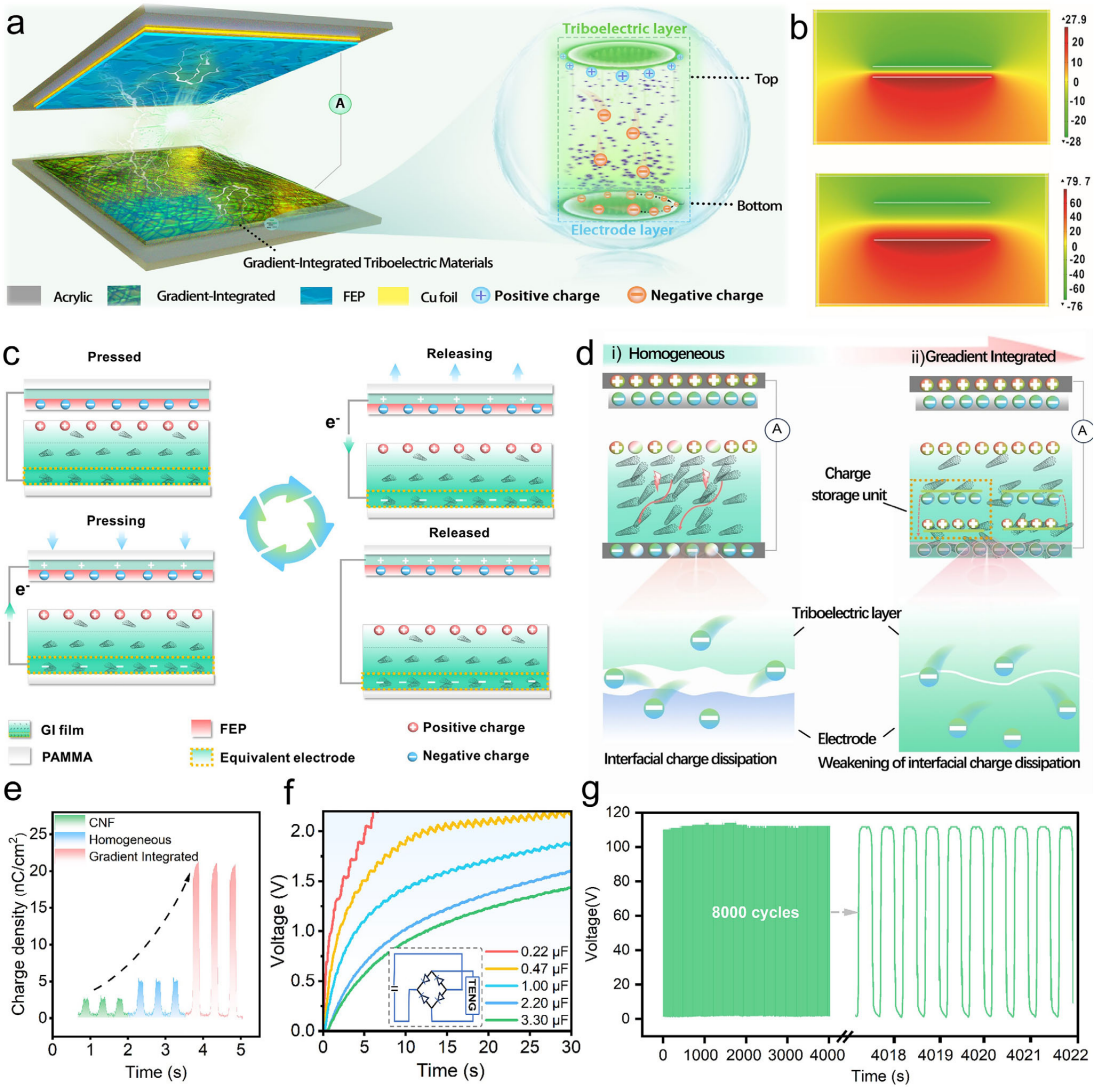
Triboelectric properties of GI triboelectric materials. (a) Schematic of the TENG assembly. (b) COMSOL simulation of potential distribution during contact separation. (c) Charge transfer mechanism. (d) Charge retention mechanism. (e) Surface charge density comparison of the three materials. (f) Capacitor charging by the GI material. (g) Output stability over cycles.

To optimize the output performance, uniformly doped films with varying MWCNT contents were first evaluated (Figure S8). The output voltage initially increased and then decreased with increasing MWCNT content, peaking at approximately 70 V at 10 wt.% MWCNT. Specifically, uniform doping increased the open‐circuit voltage (*V*_OC_) from 24 to 70 V and the short‐circuit current (*I*_SC_) from 2.5 to 4.5 µA (Figure S9).

To decouple the contributions of gradient distribution and electrode integration, composition‐ and thickness‐matched control samples were prepared. A homogeneous film and a gradient film (GI‐1/3/6‐E) with identical total MWCNT loading (10 wt.%) and total thickness (∼ 295 µm) were tested under the same conditions (Cu/Ni electrodes, 2.25 cm^2^, 60% RH, 10 kPa). The gradient film exhibited a higher surface charge density than the homogeneous film (Figure S10), confirming the contribution of gradient distribution to charge trapping and output.

Different gradient ratios (1/3/4, 1/3/6, 1/3/8, and 1/3/10) were further evaluated. The surface charge density initially increased and then decreased with increasing gradient ratio, reaching a maximum at 1/3/8 (Figure S11a). At this ratio, the *V*_OC_ and *I*_SC_ of the GI‐1/3/8 film reached 110 V and 8.0 µA, respectively (Figure S9). This is attributed to the moderate gradient facilitating charge trapping in the T‐CNF‐rich top layer and charge transport in the MWCNT‐rich bottom layer; an excessive gradient leads to increased leakage current and dielectric loss, while an insufficient gradient restricts charge transport. To isolate the effect of electrode integration, samples with and without external electrodes were compared at the 1/3/8 ratio. The surface charge density without external electrodes (20.5 nC cm^−2^) was 76.7% higher than that with external electrodes (11.6 nC cm^−2^), indicating that the integrated design suppresses interfacial charge dissipation and transport loss (Figure S11b).

By optimizing the gradient structure, the surface charge density increased from 2.2 nC cm^−2^ (pure T‐CNFs) to 5.0 nC cm^−2^ (uniformly doped film) and then to 20.5 nC cm^−2^ (GI film), reaching 931.8% of that of pure T‐CNFs, demonstrating the superior charge generation and retention capability of the GI material (Figure 5e). A TENG assembled from the GI material charged a 0.22 µF capacitor to 2 V within 5 s (Figure 5f), confirming its power supply capability for self‐powered sensing. The durability test results demonstrate that the TENG output remains stable after 8,044 contact‐separation cycles (Figure 5g), confirming that the gradient structure enhances long‐term durability and stability. Additionally, evaluation of its electrical output performance over a temperature range of 60°C–110°C shows that the material possesses excellent high‐temperature stability, thereby significantly broadening the application scope of triboelectric materials under diverse environmental conditions (Figure S12).

### 2.6 GI triboelectric Materials for Motion Detection

For wearable data collection, AI‐based dynamic monitoring using distributed sensors enhances physical activity assessment [40]. The GI triboelectric material possesses excellent mechanical strength and resists slippage caused by dielectric‐triboelectric layer mismatch under prolonged mechanical training, making it suitable for high‐intensity, long‐term motion applications (Figure 6a). A 1D convolutional neural network (1D CNN) is employed to train on single‐channel Morse code voltage signals, extracting temporal features for letter classification. The model outputs a probability distribution for each letter and assigns the class with the highest probability as the recognition result (Figure 6b). Attached to the elbow joint, the sensor accurately recognizes coded signals such as “SOS” and “GXU” (Figure S13 and Figure 6c). The confusion matrix (Figure 6d) shows 99.0% accuracy in distinguishing different letters, confirming system reliability.

**FIGURE 6:**
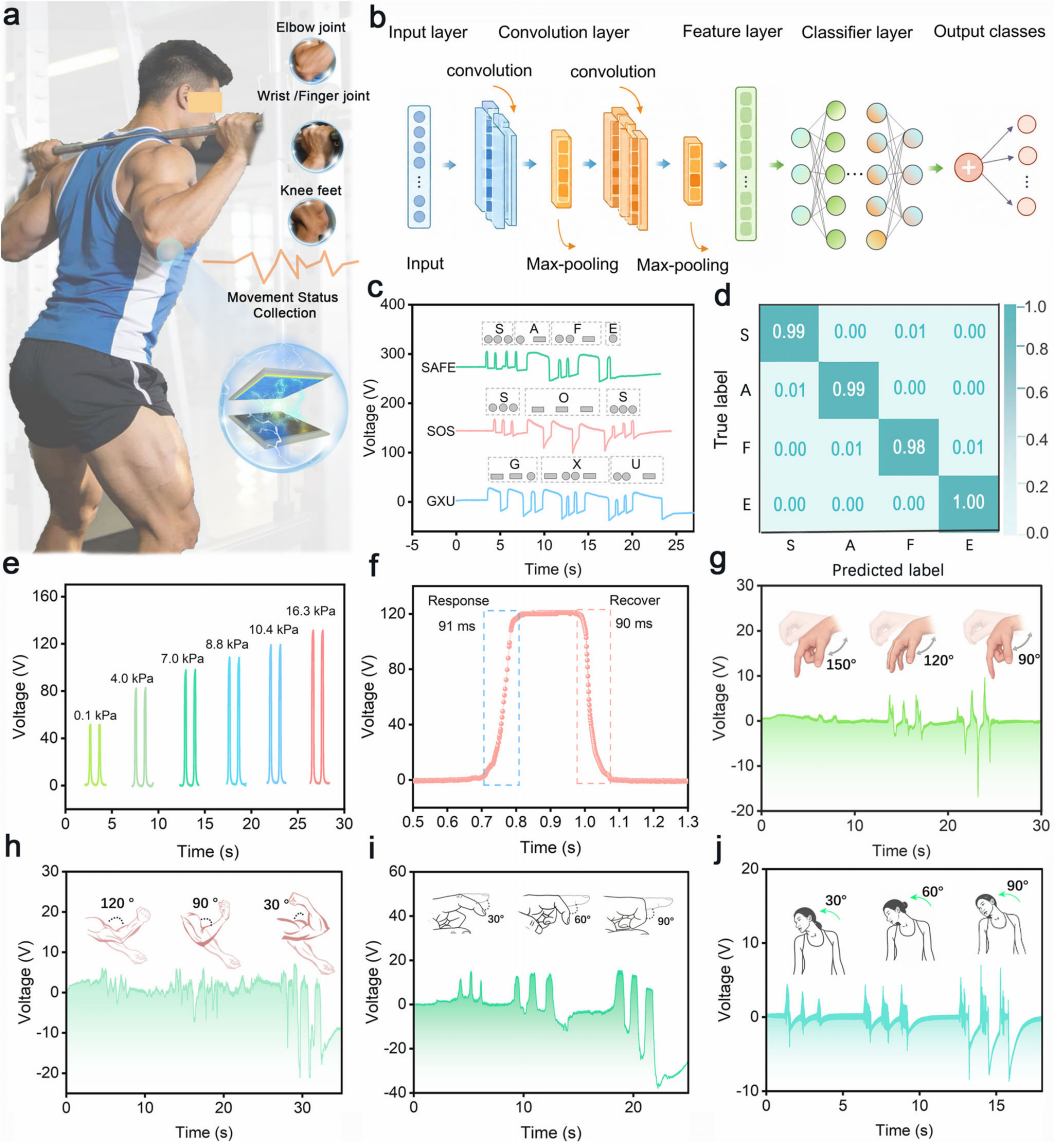
Application of GI triboelectric materials in pressure signal detection. (a) Schematic of integrated triboelectric materials for posture control during exercise. (b) Schematic diagram of a convolutional neural network. (c) Signal encryption examples for “SAFE,” “SOS,” and “GXU”. (d) Confusion matrix for the Morse code “SAFE”. (e) Electrical output under different pressures. (f) Signal response and recovery time. (g–j) Accurate detection of finger, elbow, and neck bending angles using integrated triboelectric sensors.

The GI triboelectric material exhibits significant signal variations under different pressures (0.1–16.3 kPa) (Figure 6e). When the GI sensor is pressed with the fingers at different pressures, it produces distinct signal responses; integrating it into the sole of a shoe allows for precise differentiation between various motion states (Figure S14). It exhibits a relatively fast response to motion signals, with response and recovery times of 91 and 90 ms, respectively, demonstrating the ability to capture fast motion (Figure 6f). When integrated into the wrist, it can accurately detect the angle of wrist flexion (90°–150°) based on signal variations (Figure 6g). When further integrated into the arms, fingers, and neck, the waveforms from the soles of the feet demonstrate real‐time, continuous monitoring capabilities, with clear, noise‐free signals that can accurately identify different postures and degrees of flexion. This technology shows tremendous potential in rehabilitation training, sports performance analysis, and human‐computer interaction (Figure 6h–j).

## 3 Conclusion

Inspired by the gradient architecture of sea urchin spines, we fabricated a gradient‐integrated (GI) cellulose triboelectric material with a continuous concentration gradient via vacuum‐assisted layer‐by‐layer filtration. This design creates a non‐uniform electric field within the material, inducing microcapacitive charge‐storage units that enhance charge trapping. Meanwhile, the gradient interface optimizes the compatibility between the triboelectric layer and the electrode layer, suppresses interfacial charge dissipation, and provides a low‐impedance charge‐transport pathway. Benefiting from these synergistic effects, the GI material simultaneously achieves high surface charge density, excellent cycling stability, significantly enhanced mechanical strength and toughness, as well as good water and thermal stability. Furthermore, the integrated interfacial architecture effectively overcomes the weak adhesion and interfacial slippage commonly observed between conventional triboelectric layers and electrodes, substantially improving the environmental adaptability and long‐term reliability of cellulose‐based triboelectric materials. This work not only resolves the inherent conflict between charge trapping and dissipation in conventional doping strategies but also alleviates the interfacial mismatch in cellulose‐based TENGs, offering new insights for interfacial design and functional integration in sustainable wearable electronics.

## 4 Experimental Methods

### 4.1 Materials and Chemicals

The TEMPO‐oxidized cellulose nanofibers (T‐CNFs) used in this study were supplied by Tianjin Mu Jingling Biotechnology Co., Ltd. The T‐CNFs had a solid content of 1.12%, a purity greater than 99%, and a length of 0.8–2.0 µm. The sand‐core funnel was purchased from Beijing Beibo Bomei Glass Co., Ltd. It had a pore size of 2–4 µm, a diameter of 100 mm, and a capacity of 500 mL. The microporous filter film was purchased from Tianjin Jinteng Laboratory Equipment Co., Ltd. It is made of a water‐based (mixed fiber) material, with a pore size of 0.22 µm and a diameter of 100 mm.

### 4.2 The Preparation of T‐CNFs Films

Weigh 50 g of cellulose gel with a solid content of 1.12 wt.%. Disperse 50 g of T‐CNFs in 300 g of water to prepare a 0.16% T‐CNFs solution. Sonicate the T‐CNFs solution at 800 W for 15 min, then stir it at 500 rpm on a magnetic stirrer at room temperature for 12 h. Using a mixed cellulose (MCC) filter membrane with a pore size of 0.22 µm, the cellulose suspension was filtered using a sand‐core funnel (G5). After vacuum filtration to obtain the T‐CNFs film, it was placed in a plate vulcanizer and hot‐pressed at 50°C for 6 h, then dried in an oven at 50°C for 24 h.

### 4.3 Preparation of Uniformly Doped Films

The T‐CNFs suspension was prepared as described above. Graphitized multi‐walled carbon nanotubes (MWCNTs) were added to the T‐CNFs suspension at loadings of 1, 5, 10, 20, 30, and 40 wt.% relative to the oven‐dried weight of T‐CNFs. The mixture was first ultrasonicated (100 W) under magnetic stirring for 30 min, and then magnetically stirred at 500 rpm for 6 h to ensure homogeneous dispersion. Following the same filtration procedure, the mixture was vacuum‐filtered to form T‐CNFs/MWCNT composite films. The resulting films were hot‐pressed at 50°C for 6 h in a plate vulcanizer and subsequently dried in an oven at 50°C for 24 h.

### 4.4 Preparation of GI Films

Gradient multilayer T‐CNFs/MWCNT films were fabricated via layer‐by‐layer vacuum filtration self‐assembly. The gradient ratio x/y/z (MWCNT/T‐CNFs mass ratio in top, middle, and bottom layers) was 1/3/4, 1/3/6, 1/3/8, and 1/3/10, denoted as GI‐1/3/4, GI‐1/3/6, GI‐1/3/8, and GI‐1/3/10. The corresponding MWCNT loadings (top/middle/bottom) were 3/9/12, 3/9/18, 3/9/24, and 3/9/30 wt.%, based on the oven‐dried T‐CNFs weight in each layer. Total MWCNT loadings (average of three layers) were 8, 10, 12, and 14 wt.%. The T‐CNFs mass per layer was constant to ensure a well‐defined gradient. The mixtures were first ultrasonicated (100 W) under magnetic stirring for 30 min and then magnetically stirred at 500 rpm for 6 h to ensure homogeneous dispersion. Following the same procedure, the mixtures were sequentially vacuum‐filtered in descending order of concentration to construct the gradient composite films. The resulting films were hot‐pressed at 50°C for 6 h in a plate vulcanizer and subsequently dried in an oven at 50°C for 24 h.

### 4.5 GI‐TENG Test Assembly

The gradient‐integrated composite film with a low MWCNT concentration (top layer) is used as the triboelectric layer, while the layer with a high MWCNT concentration (bottom layer) serves as the electrode layer. For the other electrode, FEP acts as the triboelectric layer and conductive fabric tape as the electrode layer. The two electrodes are connected via copper wires to form a closed circuit. The TENG is assembled using a vertical contact‐separated configuration and tested using a linear motor drive. The electrical signals generated by the external circuit were collected by a data acquisition card and converted by a program running on LabVIEW software. The test area measured 1.5 × 1.5 cm^2^ (Further details on the GI films are provided in Table S1). The cyclic durability test was performed at a contact‐separation frequency of 2 Hz and an applied pressure of 10.4 kPa, with an ambient humidity of 55% RH at room temperature (25°C).

### 4.6 Characterization

The Fourier transform infrared spectrometer was used to analyze the chemical structure, with a wavelength range of 500–4000 cm^−1^. The resolution was set to 0.4 cm^−^^1^ during the characterization of the material's chemical structure. X‐ray diffraction was used to analyze the crystal structure of the thin film, with a scanning range of 2*θ* = 5°–80° and a scanning speed of 3° min^−1^. X‐ray photoelectron spectroscopy (XPS) was used to perform chemical elemental analysis of the materials. The test parameters were set to a voltage of 14 kV and a current of 7 mA. The qualitative analysis of chemical bond interactions was performed using a laser Raman spectrometer (Raman) with an excitation wavelength of 532 nm and a sample measurement wavelength range of 800–3000 cm^−1^. Thermogravimetric analysis (TG) of the materials was performed using a STA 449 F5 Jupiter synchronous thermal analyzer manufactured by NETZSCH (Germany), with a testing temperature range of 30°C–800°C and a heating rate set at 10°C min^−1^. A FLIR thermal camera (FLIR, USA) was used to record the temperature changes of the samples uniformly heated to 25.9°C with a small LED bulb. The sample thickness was kept at approximately 295 µm under ambient conditions of 25°C and 50% RH. The cooling rate within 30 s was defined as (*T*_0_ – *T*_30_)/*T*_0_ × 100%. Mechanical property testing of the composite materials was conducted using a universal testing machine (03050302) from the United States. The tensile speed was set to 10 mm min^−1^. Surface potential was measured using a Kelvin probe force microscope (KPFM). The hydrophilicity and hydrophobicity of the materials were measured using a Luex contact angle measuring instrument (DSA100, Germany). The resistivity of the composite materials was tested using a resistivity tester. Based on the relationship *σ* = 1/*ρ*, where *σ* is the conductivity (µS cm^−1^) and *ρ* is the resistivity (MΩ·cm), the conductivity of each material was calculated. The dielectric constant and dielectric loss of the materials were measured using an impedance analyzer to determine the dielectric constant and dielectric loss of the samples in the frequency range of 10^2^–10^6^ Hz. An electrometer (Keithley 6514, USA) and a data acquisition card (NI‐USB6259, USA) were used to record the output electrical signals in real time. The test conditions were 25°C and 50% relative humidity. Using COMSOL Multiphysics 6.3, a two‐dimensional axisymmetric transient model of the contact‐separation triboelectric nanogenerator was established by coupling the Solid Mechanics Module and the Electromagnetics Module. COMSOL Multiphysics software was used to model the electric field distribution in uniformly doped and gradient‐doped materials, with the external electric field controlled at 880 kV m^−1^.

## Author Contributions

**Pinle Zhang**: conceptualization, methodology, software, visualization, data curation, Writing – review and editing, Writing – original draft. **Yanhao Xu**: conceptualization, methodology, data curation, software, validation, writing – original draft. **Huancheng Huang**: software, data curation, formal analysis. **Neng Xiong**: formal analysis, supervision, project administration. **Xinke Yu**: project administration, supervision. **Xinyue Nong**: data curation, investigation. **Yingping He**: formal analysis. **Ruizhi Li**: supervision. **Shuangfei Wang**: resources, funding acquisition. **Xinliang Liu**: writing – review and editing, resources, funding acquisition, project administration.

## Conflicts of Interest

The authors declare no conflicts of interest.

## Supporting information




**Supporting File 1**: advs78778‐sup‐0001‐SuppMat.docx.

**Supporting File 2**: advs78778‐sup‐0002‐VideoS1.mp4.

## Data Availability

The data that support the findings of this study are available from the corresponding author upon reasonable request.
