# Supplementary material for: Gradient‐Integrated High‐Strength and High‐Toughness Cellulose Triboelectric Materials for Enhanced Charge Storage and Transport
Source: Adv Sci (Weinh). 2026 Nov 10:e78778. Online ahead of print. doi: 10.1002/advs.78778 (PMC13752247; doi:10.1002/advs.78778)
Supplement: Supplementary file 1 [file ADVS-9999-e78778-s002.docx]

Supplementary information

**Gradient-Integrated High-Strength and High-Toughness Cellulose Triboelectric Materials for Enhanced Charge Storage and Transport**

*Pinle Zhang, ^1, #^ Yanhao Xu, ^1, #^ Huancheng Huang**,* *^1^ Neng Xiong, ^1^ Xinke Yu, ^1^ Xinyue Nong, ^1^ Yingping He,* *^1^ Ruizhi Li,* *^1^ Shuangfei Wang,* *^1^ Xinliang Liu*, ^1^*

*1Guangxi Key Laboratory of Clean Pulp & Papermaking and Pollution Control, School of Light Industry and Food Engineering, Guangxi University, Nanning 530004, China*

**Corresponding Author: E-mail:* *xinliang.liu@hotmail.com*

**Figure S1.** The cross-sectional morphology of the 24%, 9%, and 3% MWCNT-doped layers, magnified.

.

**Figure S2.** High-resolution C 1s XPS spectrum of the T-CNFs film.

**Figure S3.** Tensile stress-strain curves of MWCNT films at different doping ratios.

**Figure S4.** Changes in the contact angle of the three materials over 0–60 seconds.

**Figure S5.** TG curves for three materials.

**Figure S6.** Cyclic voltammetry (CV) curves of the sample at different scan rates (10, 50, 100, 500, 1000 mV·s⁻¹).

**Figure S7.** The schematic diagram of charge transfer between FEP and GI film during the contact-separation process.

**Figure S8.** Triboelectric open-circuit voltage of MWCNTs at different doping levels.

**Figure S9.** Triboelectric properties of the three materials. (a) Open-circuit voltage; (b) Short-circuit current.

**Figure S10.** Comparison of surface charge density between homogeneous and gradient (GI-1/3/6) films with Cu/Ni electrodes at a MWCNT loading of 10 wt.% relative to the T-CNFs solid content.

**Figure S11.** (a) Surface charge density of GI films with different gradient ratios (1/3/4, 1/3/6, 1/3/8, and 1/3/10); the maximum surface charge density is obtained at 1/3/8. (b) Comparison of the electrical output performance with and without external electrodes at the 1/3/8 ratio.

**Figure S12.** The electrical output remains stable between 60 and 110°C.

**Figure S13.** International Morse code chart.

**Figure S14.** The integrated triboelectric sensor accurately detects the pressure and motion status of different fingers.

**Table S1.** Formulation, structural parameters, and electrode configurations of homogeneous and gradient T-CNFs/MWCNT composite films with different gradient ratios.

| Sample | Structure | Total MWCNT loading | Gradient ratio | No. of layers | Layer thickness | Electrode | Corresponding figure |
| --- | --- | --- | --- | --- | --- | --- | --- |
| HO | Homogeneous | 10 wt% | - | 1 | 295.0 μm | With | Fig.S10 |
| GI-136-E | Gradient | 10 wt% | 1/3/6 | 3 | 92.0/97.4/105.3 μm | With |  |
| GI-134 | Gradient | 8 wt% | 1/3/4 | 3 | 92.0/97.4/100.0 μm | Without | Fig.S11 |
| GI-136 | Gradient | 10 wt% | 1/3/6 | 3 | 92.0/97.4/105.3 μm | Without |  |
| GI-138 | Gradient | 12 wt% | 1/3/8 | 3 | 92.0/97.4/107.9 μm | Without |  |
| GI-1310 | Gradient | 14 wt% | 1/3/10 | 3 | 92.0/97.4/110.6 μm | Without |  |
| GI-138-E | Gradient | 12 wt% | 1/3/8 | 3 | 92.0/97.4/107.9 μm | With |  |
